# Neonatal cross-infection due to *Listeria monocytogenes*

**DOI:** 10.1017/S0950268822000504

**Published:** 2022-03-18

**Authors:** J. McLauchlin, C. F. L. Amar, K. A. Grant

**Affiliations:** 1 UK Health Security Agency, Food Water and Environmental Microbiology Services, National Infection Service, Colindale, London NW9 5EQ, UK; 2 UK Health Security Agency, Gastrointestinal Bacteria Reference Unit, National Infection Service, 61 Colindale Avenue, London NW9 5EQ, UK

**Keywords:** *Listeria monocytogenes*, listeriosis, neonatal cross-infection

## Abstract

Neonatal listeriosis is rare and detecting more than one case together would be unlikely without a causal link. Thirty-five instances of neonatal listeriosis where cross-infection occurred in the UK and Ireland were reviewed together with 29 other similar episodes reported elsewhere. All instances comprised an infant who was ill at or within one day of delivery and who had direct or indirect contact with a second infant, or in the minority, two or more infants, who then usually developed meningitis 6 to 12 days later. In most instances, the infants were nursed on the same day in obstetric units or new-born nurseries and consequently, staff and equipment were common: hence, the likely route of transmission was via direct or indirect neonate to neonate contact. In one instance, a stethoscope was used on both infants nursed in different parts of the same hospital. In a further incident, the mother of the early-onset infant cuddled a baby from an adjacent bed who developed meningitis 12 days later. The largest outbreak occurred in Costa Rica where nine neonatal listeriosis cases resulted after bathing in mineral-oil shortly after birth which had been contaminated from the early-onset index case.

## Introduction

Listeriosis is an infection caused by the bacterium *Listeria monocytogenes* and the disease was first described in detail occurring amongst laboratory animals [1]. It is now clear that listeriosis is an important disease for humans and, although rare, presents as a serious systemic infection [2]. The disease is predominantly foodborne, most often affecting the vulnerable including those over 60 years of age, the immunocompromised and pregnant females with their unborn or new-born infants [2]. Listeriosis is the most severe foodborne infection reported in the European Union in terms of death and hospitalisation [3]. Evidence of foodborne listeriosis in England and Wales between 1981 and 2015 was previously reviewed [4], cases occurring either sporadically or as small clusters, with just one large foodborne outbreak of 378 cases. However, not all cases of human listeriosis are directly attributed to eating contaminated food: the widespread distribution of *L. monocytogenes* in the environment provides numerous potential ways for transmission. Direct contact with the environment was described in a single case of listeriosis occurring in London in 1997 where a 37-year-old male developed a fever and septic arthritis of the knee following a graze to the same knee while swimming in an open-air swimming pool. *L. monocytogenes* was detected both in synovial fluid from the infected knee and the blood of this patient [5]. Furthermore, cutaneous or ocular listeriosis resulting from contact with infected animals or animal material has been described [6–8].

Listeriosis is transmitted from the pregnant woman to her unborn infant who either dies *in utero* or is born with severe systemic infection. The first description of human listeriosis during the neonatal period was by Burn in the USA [9]. Amongst a series of four neonatal listeriosis cases, two infections were described from infants who were born on the same day in 1934 in the same hospital. The first case was premature and ill at delivery: *L. monocytogenes* was cultured from this infant's blood. The second case was born apparently healthy and became unwell on the 8th day after deliver and died 6 days later: *L. monocytogenes* was cultured from the infant's blood and CSF. Although the possibility of cross-infection was not discussed by Burn [9], neonatal cross-infection is a likely scenario. Ten episodes of neonatal cross-infection were previously reviewed which occurred in the UK between 1971 and 1984 [10]. These cases showed a common pattern, similar to that described by Burn [9] of a congenitally infected infant who was recognised as ill at birth and who had direct or indirect contact with an apparently healthy second infant who developed meningitis 8–12 days later.

The purpose of this report is to provide a reminder of listeriosis as a cause of neonatal cross-infection and to review a larger series of 35 instances which occurred in the UK and Ireland. In addition, 29 similar instances reported elsewhere in the peer-reviewed literature by others are reviewed.

## Materials and methods

The neonatal period was defined as commencing on the birth date and ending 28 complete days after birth (NHS Data Model and Dictionary 2021, available from https://datadictionary.nhs.uk/nhs_business_definitions/neonate.html). Data on cases of human neonatal listeriosis in the UK and Ireland between 1967 and 2019 were considered from within records held by UK Health Security Agency (UKHSA) or its predecessor organisations.

A case of neonatal listeriosis was defined as an illness clinically compatible with a diagnosis of listeriosis within the neonatal period with the isolation of *L. monocytogenes,* usually from blood and/or CSF. A mother and her unborn or newly delivered infant(s) were considered as a single case. All secondary (late onset) neonatal cases were considered each as single cases. Reporting for the UK was voluntary until 2010 when The Health Protection (Notification) Regulations came into force and reporting of all human listeriosis cases became mandatory (http://www.legislation.gov.uk/uksi/2010/659/pdfs/uksi_20100659_en.pdf).

Cultures of *L. monocytogenes* were voluntarily sent to the national reference laboratory within UKHSA. Typing methods in the UK series (serotyping, phage typing, amplified fragment length polymorphisms and fluorescent amplified fragment length polymorphisms) were applied as outlined previously [4]. Whole-genome sequencing (WGS) was performed as outlined previously for confirmation of identity and characterisation [11] and was applied to all cultures received from 2015 together with selected cultures prior to 2015. Clonal complexes (CCs) were derived from WGS analysis with the designation of the Institut Pasteur international MLST database for *L. monocytogenes* designation (http://bigsdb.pasteur.fr/listeria/listeria.html). Pairwise comparisons of single nucleotide polymorphism (SNP) distances were performed between isolates from cases: isolates of *L. monocytogenes* linked within a 5 SNP single linkage cluster were considered to be of common origin with each isolate having ≤5 SNPs difference with at least one other isolate within that same cluster. Sequence reference numbers from the cultures described in this study are deposited to the Short Read Archive (BioProject PRJNA248549) and are available from https://www.ncbi.nlm.nih.gov/bioproject/?term=PRJNA248549.

Case-reports in the peer-reviewed literature from other countries were identified through Pubmed searches using the search criteria: ‘listeriosis’, ‘*Listeria*’, ‘neonate’, ‘neonatal’, ‘cross-infection’, ‘nosocomial’. Further reports were located through reference citations in case-reports identified above.

## Results and discussion

### Cases in the UK and Ireland

There was a total of 35 instances of possible neonatal cross-infection which occurred between 1971 and 2012: ten episodes have been described previously as case reports [12–21].

Three of these instances occurred in Scotland, two in Wales two in Ireland and the remaining 28 occurred in England. Twenty-three of the instances occurred between 1971 to 1989, six between 1990 and 1998 and the remaining six between 2006–2012. There were no records of neonatal cross-infection identified before 1971 or after 2012.

All 35 instances showed a common pattern (Table 1) of a congenitally infected infant who was recognised as ill at birth or within one day of delivery. Within each episode, there was direct or indirect contact in the same hospital with an apparently healthy second infant (33 episodes), and in two instances two infants (one of which were twins where both became infected [20]). In all instances, the late-onset cases developed meningitis and were diagnosed as ill between 2 and 18 days after contact with the early onset case (50% between 6 and 12 days, mean 7.7 days). In 20 of the 35 instances, the infants were nursed on the same day in the obstetric unit, recovery room or new-born nursery, consequently staff or equipment were common. The most common equipment noted was the use of the same resuscitaire in five instances (Table 1).

**Table 1.** Features of 35 episodes of neonatal cross-infection occurring in the UK and Ireland 1971–2015

Features	Numbers of neonatal cases	
	Index case, *n* = 35 (early-onset)	Secondary cases, *n* = 37 (late-onset)
Numbers of cases per episode	35 with 1 case per episode	33 with 1 case per episode 2 with 2 cases per episode
Onset of symptoms (days after birth)	26 at birth 1 within one day 8 NR	1 at 2 days 3 at 3 days 2 at 4 days 1 at 5 days 4 at 6 days 3 at 7 days 4 at 8 days 3 at 9 days 3 at 10 days 2 at 12 days 1 at 16 days 1 at 18 days 9 NR
Isolation of *L. monocytogenes*:		
Maternal sites	8 HVS 1 HVS and breast fluid 1 blood 1 NR	
Infant sites	1 CSF 14 blood 2 blood and placenta 6 blood and surface swabs 1 PM lung 1 GA and conjunctiva 5 surface swabs 1 rectal swab 3 NR	18 CSF 4 CSF and blood 9 blood 1 chest drain 5 NR
Contact	35 born in the same hospital 7 born in the same delivery room or nursed in the same SCBU or postnatal ward 20 Born on the same day in the same obstetric unit, recovery room or new-born nursery 3 episodes one-day apart, 1 two-days, 2 five days, 1 seven days, 1 ten days apart 8 episodes with common equipment: 4 same resuscitaire, 2 same weighing scales, 1 same rectal thermometer, 1 same stethoscope	

CSF, cerebrospinal fluid; GA, gastric aspirate; HVS, higher vaginal swab; NR, not recorded; PM, post-mortem; SCBU, special care baby unit.

Seven of the episodes occurred where common contacts extended over time periods longer than one day, the longest being where the same delivery room which was used by what became the late-onset case 10 days after the birth of the early-onset case.

Two of the episodes differed in the likely route of transmission. In the first, the late-onset case was born 9 h before the index case but did not share the same cot or geographical location within the hospital at any time. Both infants were attended by the same medical practitioner who attended the early-onset case who was born by the emergency caesarian section, and the practitioner only examined the late-onset infant prior to initial discharge on the same day as their delivery. The only common factor was the medical practitioner: it was suggested that the most likely mode of transmission was the use of the same stethoscope, although the bacterium was not recovered from this instrument. The late-onset case was readmitted 10 days later when the bacterium was recovered from a CSF sample [21]. *L. monocytogenes* was recovered from the placenta and maternal HVS, but not from the early onset case as specimens were collected after the commencement of antimicrobial treatment [21]. In the second instance, the congenitally infected infant died at birth and 5 days later the mother of this infant was nursed in an open postnatal ward and allowed to cuddle the baby in the adjacent bed who developed meningitis at 12 days later [13].

In all 35 episodes, microbiological confirmation of listeriosis was by the isolation of *L. monocytogenes*. The bacterium was isolated from 34 of the early onset cases (22 from blood) and all of the resulting late-onset neonatal cases (22 from CSF and 10 from blood). *L. monocytogenes* was isolated from 11 of the mothers of the congenitally infected infants (HVS, breast fluid and/or blood) but never from the mothers of the late-onset cases. *L. monocytogenes* was not isolated from any environmental sites associated with any of the instances.

Of the 35 incidents, five were due to *L. monocytogenes* serogroup 1/2, 29 were due to serogroup 4 and the serogroup was not available in the final incident. In addition to serogrouping, phage typing was used between 1971 and 2001 and amplified fragment length polymorphism analysis between 2002 and 2015: within each individual instance, *L. monocytogenes* isolates from the early and late-onset cases, and, where available, the mother of the early onset case were of the same type.

WGS was applied to isolates from cases of episodes occurring between 1989 and 2012: one or more isolate was available from eight of the episodes, three were due to *L. monocytogenes* CC1, three to CC2, one to CC6 and one to CC18. Isolates from the early and late-onset neonatal cases were available from four of the episodes (including that described by Fullerton *et al*. [21]), two were CC1, one was CC2 and one CC6. Analysis of sequence data showed that, within each incident, all were indistinguishable (≤5 SNPs) and therefore indicative of a common source. Sequence accession numbers from the four episodes are SRR16976073 and SRR16976080; SRR16976074 and SRR16976072; SRR17120522 and SRR16941085; SRR16286915 and SRR16286914.

### Neonatal listeriosis reported in peer-reviewed literature

Case reports from a further 29 instances were identified from outside of the UK and Ireland occurring between 1936 and 2013. The instances occurred in 11 different countries: ten in France [22–28], three in Germany [29–31], three in the USA [9, 32, 33], two in Canada [34, 35], two in Israel [36, 37], four in Sweden [38–40], and one each in Costa Rica [41], Chile [42], Italy [43], Kuwait [44] and Spain [45]. A summary of these 29 episodes is shown in Table 2.

**Table 2.** Features of 28 neonatal cross-infection episodes occurring outside the UK and Ireland and reported in the peer-reviewed literature 1936–2013

Features	Numbers of neonatal cases	
	Index case *n* = 29 (early-onset)	Secondary case(s) *n* = 66 (late-onset)
Numbers of cases per episode	28 with 1 case per episode 1 with 2 cases per episode	14 with 1 case per episode 6 with 2 cases per episode 4 with 3 cases per episode 2 with 4 cases per episode 1 with 5 cases per episode 1 with 6 cases per episode 1 with 9 cases per episode
Onset of symptoms (days after birth)	28 at birth 2 NR	2 at 2 days 8 at 3 days 3 at 4 days 9 at 5 days 10 at 6 days 6 at 7 days 5 at 8 days 6 at 9 days 2 at 10 days 2 at 11 days 2 at 12 days 1 at 13 days 2 at 14 days 1 at 16 days 1 at 18 days 2 at >28 days 4 NR
Isolation of *L. monocytogenes*:		
Maternal sites	1 endocervix 3 HVS 2 site not stated	
Infant sites	3 CSF 1 CSF and blood 1 CSF, blood and gastric aspirate and rectal swab 5 blood 4 blood and surface swabs 1 blood and conjunctiva 1 blood and PM lung 1 PM liver and spleen 1 amniotic fluid and surface 1 bronchial aspirate and nasopharynx 2 surface swabs 1 gastric aspirate and surface 1 placenta. lochia and surface 1 placenta 1 not microbiology confirmed, histology (foci in liver and lungs) was consistent with listeriosis 4 NR	34 CSF 9 CSF and blood 1 CSF, blood and urine 5 blood 1 blood, gastric aspirate, nasopharynx 1 blood and faeces 1 bronchial aspirate 1 rectum 3 faeces 10 NR
Fomites	1 tape measure 1 mineral oil	
Contact	28 incidents, all infants born in the same hospital 29 incidents, all infants nursed in same hospital 11 born in the same delivery room 9 nursed in the same postnatal ward common equipment: 4 episodes same resuscitaire, 1 rectal thermometer, 1 incubator for transport to the ward, 1 mineral oil Maximum duration of contacts per episode: 12 same day, one episode one-day apart, one two-days, one three-days, one four-days, three five-days, one nine-days, one ten-days, one 11 days, one 14 and one 20 days.	

CSF, cerebrospinal fluid; GA, gastric aspirate; HVS, higher vaginal swab; NR, not recorded; PM, post-mortem.

The 29 episodes showed many similarities to those already described for episodes in the UK and Ireland. For each instance, infants within each episode were nursed within the same hospital. In this series, the late-onset cases were diagnosed as ill between 2 and 48 days after contact with the early onset case (48% between 6 and 12 days, mean 8 days). In 28 of the instances, all infants were born in the same hospital, in the final instance described by Dubois and Lefebvre [22], the primary and one of the secondary cases were born at home and subsequent contact occurred in a neonatal hospital ward. In 28 of the instances there was a single early-onset case detected, and in the one instance, two early-onset cases born on the same day in the same delivery unit [42]. Fourteen of the 28 incidents showed the most common pattern observed in the UK and Ireland of a congenitally infected index case with sepsis which resulted in a further single late-onset neonatal case. In a further 12 episodes two, three or four late-onset cases occurred (Table 2). In the final three episodes, a single early-onset case resulted in five [44], six [29] or nine [41] late-onset cases. Where information was available, all of the early onset cases were ill at birth and 67% of the late-onset cases were diagnosed between 9 and 12 days after contact with the early-onset case. Two of the late-onset cases occurred outside the neonatal period: one at 30 days in a cluster of three [26] and one diagnosed 48 days after delivery [45]. Velin *et al*. [26] reported a cluster of three late-onset cases diagnosed as ill at 7, 14 and 30 days later and who were nursed in the same hospital but in different rooms over a 5-day period with the early-onset case. In the report of Tortajada *et al*. [45], an early-onset case was nursed from birth in the same neonatal ward over a 10-day period as two newborn infants who were diagnosed with meningitis 9 and 48 days later. Staff and a nappy changing surface were common to all these infants.

Microbiological confirmation was obtained by the isolation of *L. monocytogenes* from all of the early-onset cases (13 from blood and 5 from CSF) except in one instance where a diagnosis of listeriosis was strongly supported by histological examination of the tissue taken at necropsy [36]. *L. monocytogenes* was isolated from maternal sites in six instances [24, 30, 33, 37, 42, 43]. From all of the late-onset cases, of the 56 infants where information was available, the bacterium was recovered from CSF in 44, blood in 17 and solely from other sites in 5 cases (Table 2). *L. monocytogenes* was isolated from non-clinical samples in two instances: a tape measure used in the delivery Unit in one instance [40] and from mineral oil used for bathing the infants after delivery in the largest series, see below [41].

Characterisation of the *L. monocytogenes* isolates from the clinical cases was not reported in 16 of the incidents. In the remaining 13, serogrouping was available for ten of the incidents and two were due to serogroup 1/2, the remaining eight were due to serogroup 4. Evidence of the same *L. monocytogenes* type being recovered from the mothers of the early-onset cases and environmental sites (where available) and both early and late-onset infants within each episode was obtained by: phage-typing [26, 27, 33, 34, 40, 43, 44]; multilocus enzyme electrophoresis [35, 41]; random amplified polymorphic DNA [46]; restriction fragment analysis [35, 43], plasmid analysis [43] and pulsed-field gel electrophoresis [28, 37, 45].

Possible routes of transmission were identified with common staff and equipment, particularly the use of the same resuscitaire in four instances (Table 2). In one instance, a common rectal thermometer was used which was not disinfected between infants [39]: the early-onset case was born shortly before two further infants who were diagnosed as ill with blood in their stool 3 days later. *L. monocytogenes* was isolated from the blood and CSF of the early-onset case, the faeces of the two late-onset cases, and the faeces of a further two asymptomatic infants who were born on the same and four days later [33]. The largest series occurred in Costa Rica in 1989 where a single early-onset case resulted in a further nine cases diagnosed as ill three to eight days after delivery [41]. All cases were born over a 13-day period in the same delivery room where newborn infants were bathed in mineral oil shortly after birth. There was evidence for contact between the oil and the babies' nose and mouth. The mineral oil was stored in an open container in the delivery room with no additional disinfection agents and was neither cleaned or completely emptied between refilling: the same strain of *L. monocytogenes* was detected in the index patient's clinical specimens as well as the open oil container in the delivery room.

## General discussion

Listeriosis is a rare disease and is predominantly foodborne: in 2019, 2–3 listeriosis cases per million of the general population were reported in the UK [3]. The reported incidence of neonatal listeriosis was 3.4 and 1.8 cases per 10 000 live births in the UK during 2004–2014 [47] and for the UK and Ireland 2017–2019 [48] respectively: neither of these series had any instances of neonatal cross-infection described here. Late-onset listeriosis is less common than early-onset disease: in a series of cases in the UK (2004–2014), UK and Ireland (2017–2019) as well as France (2009–2017), 1 out of 19, 1 out of 27 and 12 (6%) out of 189 neonatal cases were late-onset respectively [47–49]. Hence, detecting more than one neonatal listeriosis case in a single hospital over a short time period would be unlikely to occur without a causal link. It was previously reported that for 12 episodes occurring between 1971 and 1985, for every 10 early-onset neonatal listeriosis cases a further late-onset case occurred and that 24% of the late-onset cases were due to cross-infection [50]. In the UK, neonatal cross-infection occurred in the 2010–2020 less commonly than in the 1970s and 1980s, no instances were detected since 2012 and this may reflect the widespread use of intrapartum antibiotic prophylaxis directed against Group B streptococcus [47].

We here review 64 instances where nosocomial neonatal transmission of listeriosis was likely to have occurred. Evidence for cross-infection and not direct foodborne exposure for the late-onset cases in the incidents reviewed here are the proximity with an early-onset case; the recovery of the same strain of the bacterium (using a variety of techniques) from both infants as well as, where available, the mother early-onset neonatal case and not the mother of the late-onset case; a plausible vehicle of infection from staff, equipment, the neonatal environment or, in one instance, the mother of the early-onset case.

We did not detect any special characteristics in the *L. monocytogenes* isolates from the cases of neonatal cross-infection described here. However, we report here, for the first time, the use of WGS to confirm a clonal relationship between the *L. monocytogenes* isolates from four instances, and the availability of sequence information into the public domain allows further characterisation by others. It is of note that all of the incidents were either CC1, CC2, CC6 or CC18: it has been commented elsewhere that the clonal complexes CC1, CC2, CC4 and CC6 are responsible for two-thirds of the maternal/neonatal infections in France [51].

The neonatal cross-infection instances described here showed a common pattern of an infant born with congenital listeriosis (onset within 1 day of birth). In the same hospital, and within a short period of time, an apparently healthy (or more rarely more than one) neonate is born who typically develops late-onset listeriosis between the 5th and 12th day later. There was a greater proportion of incidents with more than one late-onset case per episode in the world literature as compared to that from the UK and Ireland, and probably reflects publication bias for larger incidents. The routes of exposure to the late-onset cases are hence most likely to be via direct or indirect neonate to neonate transmission via infected infant and their mother, as well as common equipment or hand contact from staff in the neonatal environment. Evidence from the cases described here indicates that persistence occurs in neonatal environments for at least a couple of weeks as illustrated by a secondary case occurring 10 days after using the same delivery suit as an early-onset case and, in a separate incident, by the bacterium being detected on a tape measure collected from the delivery suit [40]. There are further similarities with foodborne transmission and cross-infection in that eating contaminated food will provide a similar route of infection to putting a contaminated resuscitaire or contaminated mineral oil into an infant's mouth as described here. The bacterium survives well on fingertips, persisting, when present, at 10^4^ cfu/fingertip, even after washing with soap and water as well as chlorhexidine solution [52]. The importance of person-to-person transmission during the neonatal period is highlighted by the episode where the mother of an early-onset case was nursed in an open ward and handled a neonate from an adjacent bed who subsequently developed late-onset listeriosis [13]. During maternal infection, invasion of the pregnant uterus, including the foetus, occurs and can result in a death in-utero or the birth of an extremely ill infant. Amniotic fluid collected during the infection of two pregnant women in France was analysed by Courcol *et al*. [53] who detected *L. monocytogenes* at 10^8^ cfu/ml. Consequently, at delivery both the neonate as well as their mother will be heavily contaminated by this bacterium as well as the hands of attending staff, clothing and any equipment used. Obstetric complications and the birth of a sickly infant will necessitate a variety of standard and emergency equipment that could act as vehicles of infection.

The most common incubation periods were between 6 and 12 days with some cases as short as 2–3 days and two instances outside the neonatal period (30 and 48 days). The exposure route may affect the incubation period and Schuchat *et al*. [41], reported that this varied between 3 and 7-days post exposure to contaminated mineral oil. For the longest incubation periods, although it is not possible to exclude additional environmental exposures, Tortajada *et al*. [45], noted that no further cases were diagnosed in the individual hospital where the incident occurred. The incubation period for neonatal listeriosis may vary in the same way as adult listeriosis where incubation periods of at least 1 to 70 days have been reported [54]. It is also intriguing that asymptomatic cases were also reported following exposure to a rectal thermometer [39] and this may also have similarities to listeriosis outside the perinatal period where, despite widespread exposure, the attack rate is generally low.

Maternal listeriosis results from eating food contaminated with *L. monocytogenes* and can be transmitted to the unborn infant who presents with early-onset neonatal disease. Furthermore, foodborne outbreaks predominantly affecting pregnant women have occurred where the unborn infants were secondarily exposed to contaminated food via consumption by their mothers, and these outbreak have occurred both in the community (e.g. through contaminated Mexican style soft cheese [55]) or in hospital (e.g. sandwiches consumed during an anti-natal clinic [56]). The late-onset cases are therefore likely to be a secondary exposure to contaminated foods consumed by the mothers of the early-onset cases. One of the neonatal cross-infection incidents reviewed here in Canada [34] was secondary to the foodborne outbreak associated with the consumption of coleslaw salad [57]. Furthermore, in the UK from 1987 to 1989, 10 listeriosis neonatal cross-infection instances were identified whilst there was an ongoing nationwide listeriosis outbreak associated with eating pâté [58]. *L. monocytogenes* cultures from five of the 10 neonatal cross-infection instances were identified, as defined by serotype and phage-type, as being due to the same strain as that associated with the contaminated pâté. Pregnancy-associated listeriosis became much more common during this outbreak in the UK, possibly because pregnant women were advised to eat pâté as a source of iron (McLauchlin, unpublished). However, apart from the neonatal listeriosis cases reviewed here, other epidemiological patterns of transmission have been reported amongst neonatal listeriosis cases. Line and Cherry in 1952 [59] described two cases of listerial meningitis (onsets 6 and 12-days post-delivery) who were born two days apart in the same delivery room and clearly differs from the pattern described here.

In one of the instances reported in England, analysis by WGS, showed that not only were isolates from the two neonatal cross-infection cases identified as being identical, and thus indicative of a common source, but were also ≤5 SNPs different from isolates recovered 8 years later from two other cases in completely different hospitals: one in an 80 year old and the second in a 4 day-old baby. This observation is consistent with a common-source foodborne outbreak (albeit that a food vehicle was not identified) of three listeriosis cases with a further secondary case due to cross-infection.

Neonatal listeriosis is rare, hence clinicians will encounter this infection uncommonly. This report reviews the evidence for neonatal listeriosis resulting in cross-infection and should act as a reminder of this complication, particularly to these in managing new-born infants.

## Data Availability

The datasets used or analysed during this study are available from the corresponding author on reasonable request. DNA sequences are available from https://www.ncbi.nlm.nih.gov/bioproject/?term=PRJNA248549.
